# Human Enteric Pathogens in Eight Rivers Used as Rural Household Drinking Water Sources in the Northern Region of South Africa

**DOI:** 10.3390/ijerph17062079

**Published:** 2020-03-20

**Authors:** Natasha Potgieter, Simbarashe Karambwe, Lutendo Sylvia Mudau, Tobias Barnard, Afsatou Traore

**Affiliations:** 1 Microbiology Department, University of Venda, Private Bag X5050, Thohoyandou 0950, South Africa; karambwesim@gmail.com (S.K.); Afsatou.traore@univen.ac.za (A.T.); 2 Dean, School of Mathematical and Natural Sciences, University of Venda, Private Bag X5050, Thohoyandou 0950, South Africa; 3 Department of Environmental Health, Tshwane University of Technology, Private Bag X680, Pretoria 0001, South Africa; MudauLS@tut.ac.za; 4 Water & Health Research Center, University of Johannesburg, PO Box 524, 2006 Auckland Park, Johannesburg 2094, South Africa; tgbarnard@uj.ac.za

**Keywords:** enteric pathogens, diarrhoea, river water, rural communities, Vhembe District, water quality

## Abstract

People living in rural areas still rely on the use of environmental water that is contaminated by human and animal activities. This study assessed the occurrence of human enteric pathogens in rivers that are used by rural communities Vhembe District of South Africa as a source of drinking water covering two seasons (winter and summer) over a one-year period. Water quality was assessed using physico characteristics and indicator organisms (total coliforms, *E. coli*, *Clostridium perfringens*). Pathogens tested included bacteria (Pathogenic *E. coli*, *Salmonella*-, *Shigella*- and *Vibrio* spp.), protozoa (Cryptosporidium- and Giardia spp.), and enteric viruses (Rota-, Noro-, Entero-, and Adenoviruses) while using published molecular protocols. The results showed that the indicator bacteria counts exceeded South African drinking water quality guideline limits and pathogenic *E. coli* was detected in the samples. No *Shigella* spp. were isolated, while *Vibrio* spp. and *Salmonella* spp. were present; parasites were detected in four rivers and Enteric viruses were predominantly detected in the winter season. The results indicated the poor condition of water and the potential health risks to consumers highlighting the need for implementing river catchment management strategies for continued sustainability in these rivers.

## 1. Introduction

Various rivers in South Africa have been shown to be highly polluted due to impacts from water and land use practices in their catchments [1,2,3,4,5]. Very few studies and reports on waterborne diarrhoea in the rural communities in the Vhembe region (Limpopo province, South Africa) are available, and it is highly possible that some diarrhoea cases go unreported. Settlements in rural and peri-urban areas are sparsely distributed due to the rough terrain, and this hinders the capacity to provide a centralized drinking water system [6]. Most of the communities in rural areas in the Limpopo province of South Africa have limited access to municipal water and might resort to the use of nearby surface water sources for their daily subsistence [5,7].

Rural populations obtain water on an individual or household basis from the closest surface and ground water sources where the microbial quality is often unknown [8]. In the Vhembe District, many of the communities rely on river water sources, which are devoid of treatment, and water collected from boreholes for their domestic water needs. It has been reported that most of the river water in this area is of poor microbiological quality and unsafe for consumption [5,9,10]. In addition, households’ resort to the storing of water in different containers due to the distance of the sources and the infrequent availability of water [11]. These practices expose residents to waterborne pathogens that cause enteric diseases, such as diarrhoea, and have been reported as being responsible for the most health-related water quality problems [12]. Regardless of health education efforts, communities in the northern region of South Africa continue using unprotected sources without any form of treatment, as they perceive it as unimportant [13].

The use of untreated water for drinking and other domestic purposes, such as food preparation, washing clothes, and bathing, might be harmful to the communities [7]. Several human infections are waterborne and diseases, such as diarrhoea, are affected by fluctuations in weather and climate. In the Limpopo province, winters are usually dry, and the summers are hot with seasonal rains [5]. Dry conditions are associated with diarrhoea in children under five years of age, mainly due to the increase water storage in poor households, which leads to a higher risk of water contamination [14]. 

The prevention of water pollution requires effective and continuous monitoring of physical, chemical, and microbiological parameters to ascertain the possible risks that are associated with water from a particular source [15]. Continuous epidemiological and prevalence assessment of diarrhoeagenic pathogens in different water sources in rural and the majority of peri-urban communities will provide information to health statisticians on the prevalent strains circulating in these communities and these data will add to the knowledge of treatment effectiveness and intervention strategies. The objective of this study was to assess the prevalence of human enteric pathogens (bacteria, viruses, and parasites) in river water sources that are used for domestic purposes in rural communities of the Limpopo Province of South Africa.

## 2. Materials and Methods

### 2.1. Study Site

This study focused on eight rivers in the Vhembe District based on their proximity to human communities that use them as a source of drinking water. The water samples were collected from 10 sampling sites in three different Districts within the Limpopo Province: In the Thulamela Municipality (Tshinane river, Mutshundudi river, Sambandou river, Luvuvhu river: Mutoti site, Luvuvhu river: Mhinga site, Dzindi river, Madadzhe river); in the Mutale Municipality (Mutale river); and, in the Makhado Municipality (Nzhelele river, Luvuvhu river: Tshino site). 

### 2.2. Sample Collection

The samples were collected twice [once in the winter season (June, July) and once in the summer season (October, November)] in a one-year period during 2016. The samples were collected at the abstraction points used by communities in each river site. Clean sterile 10 L plastic drums were used to collect water samples for viral and parasite analysis (one each) and 2 × 500 mL sterile plastic containers for bacterial analysis were used to collect water samples for indicator assessments at each site. Observations that were related to human activities were noted during water collection. All of the water samples were stored on ice on route to the laboratories for analysis within 4 h. Temperature (Temp), electrical conductivity (EC), Total Dissolved Solids (TDS), dissolved oxygen (DO), and pH were measured in situ while using a Bante 900P portable multimeter (Bante Instruments; Shanghai, China).

### 2.3. Indicator Bacteria Detection

The Colilert Quanti-Tray^®^/2000 most probable number (MPN) method (IDEXX; Westbrook, ME; USA) was used according to the manufacturer’s instructions to determine the presence of *E. coli* and total coliforms. No dilutions were performed and, in cases, were the upper limit of the test was reached (>2419.6 MPN/100 mL) the data are reported as >2420 MPN/100 mL. The appropriate Quanti-Cult reference strains bacterial controls (*E. coli*, *Klebsiella pneumoniae*, and *Pseudomonas aeruginosa*) and distilled water (negative control) was used as controls in all the assessments. 

The membrane filtration method followed by culturing of the filters on m-CP agar plates were used to assess the presence of *Clostridium perfringens* in the river waters [16]. Briefly, 100 mL of water samples were heated at 60 °C for 20 min. in sterile glass conical flasks, as described by Mueller-Spitz et al. [17]. After cooling, the water was filtered using 0.45 µm pore size, 47 mm diameter cellulose acetate filters. (Merck, Kenilworth, NJ, USA). In this study, 3 mL, 10 mL, and 30 mL volumes were filtered [18]. The filters were transferred to sterile m-CP agar plates (Oxoid CM0992, pH 7.6) and anaerobically incubated at 42 °C for 24 h [19] in a jar containing AnaeroGen™ sachets (Oxoid, Hampshire, UK). Yellow colonies that turned pink on exposure to ammonium hydroxide fumes were considered presumptive *C. perfringens*.

### 2.4. Pathogenic Bacteria Identification

The isolation of *V. cholerae* from water samples was performed according to Choopun et al. [20]. A volume of 100 mL of each sample was filtered through a 0.45 µm pore size 47 mm cellulose acetate filter (Sartorius Biolab Products, Lasec, Cape Town, South Africa). The filters were enriched in 100 mL alkaline peptone water (Sigma-Aldrich, St Louis MO, USA, 2% NaCl) at 30 °C for 24 h. A volume of 10 µL of the enriched samples was streaked onto TCBS agar plates (pH 8.6, Davies Diagnostics Pty, Limited, Randburg, South Africa). The plates were incubated at 37 °C for 24 h. All the yellow and green-yellow colonies were considered as presumptive positive *V. cholerae*. 

The isolation of *Salmonella* spp. and *Shigella* spp. from water samples (100 mL) was performed while using the membrane filtration technique with 0.45 µm, 47 mm filters (Sartorius Biolab Products, Lasec). The membrane filters were submerged into 100 mL buffered peptone water (Oxoid CM0509, pH 7.2). The flasks were shaken by hand for 5 min. to mix the trapped bacteria on the filter pads with the pre-enrichment broth and then incubated at 30 °C and 37 °C for 24 h, respectively. A successive selective enrichment step in Rappaport-Vassiliadis Soya Peptone Broth (RVS) (Oxoid CM0866, pH 5.2), Nutrient broth (Sigma–Aldrich pH 7.5) and Selenite cysteine broth (SCB) (Difco, BD Product pH 7.0), accompanied by incubation at 42 °C for 48 h was performed. Thereafter, a loopful of the enriched samples was streaked on selective media; S-S agar (Difco, BD Product pH 7.0), a selective media for *Salmonella* and *Shigella* spp., and the plates were incubated at 37 °C for 24 h. 

All of the presumptive colonies for *Vibrio*, *Salmonella*, and *Shigella* were sub-cultured onto Nutrient agar and then subjected to the oxidase test (Oxidase strips, Sigma–Aldrich), API 20E test (bioMerieux Product, Quantum Biotech) and Gram stained according to the protocol that was described by Prescott [21] and Wiley et al. [22]. The isolates were preserved in Nutrient broth and then stored at −20 °C until further analysis.

The DNA extraction and mPCR protocol published by Mieta et al. [23] was used for confirmation of *Vibrio-*, *Salmonella-,* and *Shigella* species from the presumptive isolates. The DNA extraction and mPCR protocols that were published by Omar and Barnard [24] was used for the identification of pathogenic *E. coli* strains from the *E. coli* positive wells on the Quanti-Tray^®^/2000. The Biorad Mycycler Thermal cycler was used for all of the PCR reactions in a total volume of 20 µL. All of the colonies that could not be identified by the PCR protocols were then identified using the API20E kit, according to the manufacturer’s instructions (bioMerieux, Marcy I’Etoile, France).

### 2.5. Pathogenic Protozoa Detection

The samples were analyzed at a South African National Standards (SANS) accredited laboratory (Rand Water, Gauteng, South Africa), in compliance with ISO/IEC 17025. Method 1623.1, as described by the United States Environmental Protection Agency (USEPA) [25]. Briefly, each 10 L water sample was separately filtered using the PALL system with Envirochek capsules (Port Washington, NY, USA). The trapped oocysts and cysts together with extraneous materials were eluted while using 10% Tween 80 and then collected into a 50 mL centrifuge tube. The eluate was centrifuged at 1500× *g* to pellet the oocysts and cysts. The supernatant was aspirated. The oocysts and cysts were then separated from the extraneous materials while using the immunomagnetic separation technique employing paramagnetic beads (Dynabeads^®^ GC-Combo, Thermo Fisher Scientific, Gauteng, South Africa) that were conjugated to anti- *Cryptosporidium* and anti-*Giardia* antibodies. In the presence of oocysts or cysts, a paramagnetic complex is formed, which is then attracted by a magnet separating the oocysts/cysts from the extraneous materials. The beads were detached from the oocysts/cysts which were stained with fluorescently labelled monoclonal antibodies and 4′6-diamidino-2-phenyl indole (DAPI) on slides. The observation of the stained slides was done under fluorescence DIC microscopy (BW Optics, Nanjing, China) to assess the presence of oocysts/cysts. 

### 2.6. Pathogenic Viral Detection

Viruses were recovered from each of the 10 L surface water samples while using the glass wool adsorption-elution primary recovery and secondary concentration method. Briefly, the modified method that was described by Mans et al. [26] used 15 g of glass wool per column and a steel gauze grid (pore size = 1 mm^2^, 30 mm diameter) inserted between each of the three 5 g portions of the glass wool. Positively charged glass wool columns were used to capture the viruses that are generally negatively charged [27]. The negatively charged viruses, which had adsorbed to the glass wool, were eluted with 100 mL of sterile glycine-beef-extract buffer of pH 9.5 (0.3754% *w/v* glycine, 0.5% beef extract), which reverses the ionic charges of the viruses and releases them from glass wool. The pH of the eluate was adjusted to neutral with 1 M HCl. In the secondary concentration step, the 100 mL eluate was concentrated to a final volume of 20 mL in sterile phosphate buffered saline (pH 7.4, Sigma-Aldrich Co., USA) by polyethylene glycol (PEG)/sodium chloride (NaCl) precipitation, as described by Minor [28] and Vilagines et al. [29]. The recovered concentrate was stored at −20 °C until further processing. One millilitre each of all of the recovered viral concentrates were seeded with 10 µL of Mengovirus (5 × 10^5^ copies) as an extraction control and the nucleic acids were extracted while using the NucliSENS^®^EasyMAG^®^ instrument (BioMerieux, Marcy I’Etoile, France), according to the manufacturer’s instructions. The extracted nucleic acids were eluted into 100 µL, aliquoted in smaller volumes, and stored at −70 °C. Mengovirus were detected for all the reactions. 

Virus amplification were performed on three cell lines, which included the PLC/PRF/5 human hepatoma cell line (European Collection of Cell Cultures (ECACC) 85061113, Salisbury, UK) [30]; the BGM African Green monkey kidney cell line (ECACC 90092601) [31]; and, the Vero African Green monkey kidney cell line (ECACC84113001). All of the cells lines were propagated, maintained, and infected using standard cell culture procedures, as previously described [26,32,33]. The harvested cell culture suspensions (500 µL) were subjected to three cycles of freezing and thawing prior to nucleic acid extraction from 200 µL of harvested cell culture extracts while using the NucliSENS^®^EasyMAG^®^ instrument (BioMerieux), according to the manufacturer’s instructions. The extracted nucleic acids were eluted in 50 µL, aliquoted in smaller volumes, and then stored at −70 °C. For the integrated cell culture-molecular based assay, Enteroviruses were detected with a one-step real-time RT-PCR assay using the Quantitect Probe RT-PCR Kit (Qiagen, Hilden, Germany) and primers and a hydrolysis probe, as previously described [34], and the adenoviruses using primers and probes, as described by Heim et al. [35] in the TaqMan^®^ Environmental Master Mix 2.0 kit.

Commercial real-time RT-PCR assays for each enteric virus were used for the direct detection of selected human pathogenic enteric viruses in the recovered virus concentrate.

- Adenoviruses: a *rt* PCR, using TaqMan technology and primers and probes, as described by Heim et al. [35], was optimized and used for the analysis of all the samples. The molecular amplification and *rt* PCR detection of AdVs was done while using the TaqMan^®^ Environmental Master Mix 2.0 (Applied Biosystems, Foster City, CA, USA).
- Astroviruses: a commercial *rt* RT-PCR assay (KASV: ceeramTools^®^, CEERAM S.A.S., La Chapelle Sur Erdre, France), using TaqMan technology was used for the analysis of all the samples.
- Enteroviruses: the commercial *rt* RT-PCR assay (KENV: ceeramTools^®^), using TaqMan technology using the primers and probes, as described by Fuhrman et al. [34], was used for the analysis of all samples.
- Hepatitis A virus (HAV): a commercial *rt* RT-PCR assay (KHAV: ceeramTools^®^), using TaqMan technology and the primers and probes, as described by Costafreda et al. [36], was used for the analysis of all samples
- Norovirus GI: a commercial *rt* RT-PCR assay (KNVGI: ceeramTools^®^), using TaqMan technology and using the primers and probes, as described by Da Silva et al. [37] and Svraka et al. [38], was used for the analysis of all samples.
- Norovirus GII: a commercial *rt* RT-PCR assay (KNVGII: ceeramTools^®^) using TaqMan technology and the primers and probes, as described by Kageyama et al. [39] and Loisy et al. [40], was used for the analysis of all samples.
- Rotaviruses: a commercial *rt* RT-PCR assay (KRV: ceeramTools^®^) using TaqMan technology used for the analysis of all samples.
- Sapoviruses: the *rt* RT-PCR assay, using TaqMan technology and the primers and probes as described by Chan et al. [41] was used for the analysis of samples. The molecular amplification and real-time RT-PCR detection was executed using the Transcriptor First Strand cDNA synthesis kit (Roche) in conjunction with the LightCycler^®^ Taqman^®^ master kit (Roche).
- Mengovirus: a commercial *rt* RT-PCR assay (KMG: ceeramTools^®^), using TaqMan technology and the primers and probes, as described by Pintó et al. [42], was used for the detection of Mengovirus in all artificially seeded samples. If the Mengovirus or target were not detected, then RNA/DNA were diluted 1/10 and retested (dilutes out inhibitors).

## 3. Results and Discussion

Table 1 lists the various activities that were observed taking place around the river sites during this study. Many of these activities are considered to be risk factors and they could lead to the contamination of river water, such as agricultural activities, washing laundry, car washing, littering, dumping of animal blood, and domestic sewage disposal [4,5,43,44]. 

### 3.1. Assessment of Water Quality Using Physical Parameters and Indicator Bacteria

Table 2 summarises the physical and the microbial indicator data for the winter and summer seasons. The pH of water plays an important role for biological activities of microorganisms. In this study, the pH of water samples ranged from 6.79 to 8.19 in the summer and from 7.19 to 8.42 in winter. These were within the South African Water Quality guideline standards of 5.0 to 9.0 [45] for domestic use. Temperature is a key determinant of growth and survival of microbes in water and it plays an important role in their survival [46,47]. Microbial growth increases with temperature, and this might increase the problems related to taste, odour, and colour of water [48,49]. According to literature, warmer temperatures during summer go along with *Salmonella*, *Campylobacter*, or *E. coli* infections [50,51], and low winter temperatures favours viruses, such as Rotavirus and Noroviruses [52,53]. In this study, the temperature of water samples ranged from 22.9 °C to 28.3 °C in the summer and from 16.0 °C to 20.3 °C in winter. Several of the river sites had summer temperatures that were higher than the South African recommended water quality guideline standards of 18 °C to 24 °C (Table 2). 

TDS indicates the degree of salinity in a water sample and no health effects would be associated with water with TDS levels of 0–450 mg/L [45]. In this study, the TDS of water samples were well within the South African water quality guideline standards [45] and ranged from 26.8 mg/L to 142.1 mg/L in the summer and from 29.9 mg/L to 348.5 mg/L in winter. Electrical conductivity (EC) is a measure of the ability of water to conduct electricity, and this is directly dependent on the concentration of dissolved ions, which establishes a direct relationship between EC and TDS (DWAF, 1996). According to the DWAF [45], the target water quality guideline range for domestic use based on conductivity is 0–70 mS/m (0–700 µS/cm). The latest updates by SANS 241 [54] has specified the standard limits based on EC to be <170 mS/m (1700 µS/cm). In this study, the EC of water samples were well within South African water quality guideline standards [45] and they ranged from 53.3 µS/cm to 285 µS/cm in the summer and from 63.3 µS/cm to 601 µS/cm in winter. Dissolved oxygen (DO) characterizes the freshness of surface water which is directly related to the amount of oxygen dissolved. This is supported by a positive correlation between biological oxygen demand (BOD) and bacterial counts that Borade et al. [55] observed. According to DWAF [45], there are no specific guidelines in place for DO. In this study, the DO of water samples ranged from 2.04 mg/L to 9.88 mg/L in the summer and from 9.16 mg/L to 13.43 mg/L in winter. Several of the river sites during both seasons had DO readings that were higher than the South African water quality guideline recommended standards [45] of 6.0 to 8.0 mg/L (Table 3). These counts could have a health effect on humans and aquatic life in the rivers [56].

Total coliforms (TC) and *E. coli* are known as indicator organisms and the presence of TC provides an indication of other disease-causing organisms in the water source, while the presence of *E. coli* provides an indication of recent faecal contamination. The number of TC bacteria in drinking water should be less than 10 colonies per 100 mL, while the number of *E. coli* should be zero per 100 mL [45] if the water is used for drinking water, according to the South African Guidelines. The TC and *E. coli* counts in all the sites and in both seasons were far above the South African recommended guideline limits for drinking water (Table 2). The TC counts ranged between 1732 and 2420 MPN/100 mL in both seasons, while the *E. coli* counts ranged between 57.1 and 1299.7 MPN/100 mL in summer and between 12.2 and 2420 MPN/100 mL in winter. The high counts that were seen for *E. coli* in these rivers indicated a relatively high level of contamination with faecal human or animal wastes and have also been reported in other studies [4,5,9]; while, the high counts of TC bacteria indicate not only faecal waste, but also the presence of other potentially dangerous bacteria spp. in the water samples. 

The identification of bacterial isolates using the API20E test kit showed that some isolates that were isolated from the water sources were *Aeromonas-*, *Serratia-*, *Proteus-*, *Plessiomonas-*, *Enterobacter*, *Citrobacter-*, *Edwardsiella-*, *Yersinia-*, and *Biber steria* spp., which are coliform bacteria that could be harmful to humans and have been known to cause severe global outbreaks [49]. Therefore, the river water is not recommended to be used for drinking or other domestic purposes [45]. 

The South African water quality guidelines [45] recommends that no *C. perfringens* should be detected in a 100 mL water sample. In this study, *C. perfringens* counts ranged between 0 and 35 cfu/100 mL in summer and between 0 and >500 cfu/100 mL in winter. The prevalence of *C. perfringens* in water samples can be an indication of intermittent faecal contamination [49].

### 3.2. Prevalence of Pathogens 

Table 3 summarises the prevalence of pathogenic bacteria, protozoa, and enteric viruses in the eight rivers during the winter and summer seasons over a one-year survey. Several pathogenic strains of *E. coli* were detected and identified from the river sites. It must be pointed out that the PCRs were performed on mixed cultures from the Quanti-Tray^®^/2000 and, as such, conclusions cannot be drawn regarding the presence of a single or multiple pathogenic *E. coli* strains. In recent years, it has been shown that new pathogenic *E. coli* groups have emerged, such as the diffusely adherent *E. coli* (DAEC), Shiga toxin producing enteroaggregative *E. coli*, and adherent invasive *E. coli* [57], and highlights the need for the isolation and study of the pathogenic *E. coli* strains in these river samples. 

The most prevalent strains identified included atypical enteropathogenic *E. coli* (EPEC), followed by enteroaggregative *E. coli* (EAEC). EPEC has emerged as an important pathogen in outbreaks of acute diarrhoea in developed [58] and developing countries [59,60] and both EPEC and EAEC infections are dangerous in immunocompromised individuals and children [61,62,63]. A study by Traore et al. [5] on some of the rivers in the Venda region detected several pathogenic strains of *E. coli*, such as enteroinvasive *E. coli* (EIEC), enterotoxigenic *E. coli* (ETEC), and enteropathogenic *E. coli* (EPEC), which are strains that are known for causing diarrhoea in children [24] and that was also detected in this study. 

*Vibrio* spp. could cause cholera, which is responsible for high mortality, and is most commonly transmitted via contaminated water. Several communities in the northern parts of South Africa have experienced a large cholera outbreak during 2008/2009, and a total of 721 cases were confirmed. This outbreak was due to human movement, lack of sanitation infrastructures, and contaminated water sources [64,65]. In this study, the presence of *V. cholerae* was shown through the detection of the *sodB* gene [66]. This gene was detected in 9/10 (90%) and in 5/10 (50%) of the sources, respectively, during the summer and winter seasons over the study period. Nontoxic *Vibrio* spp. is widespread in water environments [49] and *V. cholerae* has been isolated from surface water [67,68] with the occurrence of *V. cholerae* in water sources that are linked to faecal pollution [69]. 

*Salmonella* spp. has been repeatedly detected in various types of natural waters, such as rivers, lakes, coastal waters, estuarine, as well as contaminated ground water [70,71,72,73]. The presence of *Salmonella* spp. in natural water resources has also been attributed to runoff from fields with animal husbandry and the disposal of untreated sewage [70]. A study by Potgieter et al. [74] on surface water used as drinking water source in the Vhembe District of Limpopo Province in South Africa has shown that the water was positive for *Salmonella. Salmonella* spp. is widespread, but certain species can cause gastrointestinal disease, septicemia, and enteric fever, and can contaminate water and food [49]. In this study, *Salmonella* spp. was detected in both seasons from all sites while using the *ipa*B gene. No *Shigella* bacteria were identified during this study.

River water has been shown to be subjected to contamination by protozoan parasites, such as *Cryptosporidium* and *Giardia,* due to point or non-point pollution sources [75]. According to Robertson et al. [76], *Cryptosporidium* and *Giardia* are both associated with sewage and, thus, contamination of water sources by sewage threatens human health due to their low infectivity dose as low as 10 oocysts/cysts [77,78]. Reports on parasitic organisms in wastewater are rare in South Africa and, yet, *Cryptosporidium* and *Giardia* are reported to be the most prevalent parasites in wastewater samples [79]. In this study, *Cryptosporidium* and *Giardia* were detected in four of the river sites. Three of the river sites tested positive for the presence of *Giardia* sists, of which the Madadzhe river site had 62 cysts/100 mL. Only the Tshihane river site tested positive for the presence of *Cryptsporidium* oocysts. 

Generally, human enteric viruses are excreted in high concentrations in the feces of infected people and they have great potential to pollute water sources [80,81]. Outbreaks caused by viruses in South Africa have been reported by Taylor et al. [82], Mans et al. [83] and Rinaldi et al. [84]. 

Similarly, only a few studies have been done and reported on the prevalence of viruses in South African water sources, such as the prevalence of Enteroviruses [27,85,86,87,88,89]; Astrovirus [90,91], Rotaviruses [89,92,93] and Hepatititis A virus [89,90,94].

Enteric viruses are mainly transmitted by faecally-contaminated water or food. Infected individuals excrete enteric viruses in numbers up to 10/g faeces and compared with most pathogens, the minimal infectious dose is extremely low. It has been reported that a single virus can cause infection [54]. In this study, viruses were detected in all river sites, except the Sambandou river site (Table 3). Direct PCR detection was more sensitive in showing the presence of viral strains in the water samples. Rotavirus, Enterovirus, and Astrovirus were only detected during the winter months supporting the report of Steele and Glass [95] that Rotaviruses and Enteroviruses prefer dry winter months. Van Heerden et al. [96] has shown that Adenoviruses are linked to respiratory and gastrointestinal infections. It is known that viruses have a low infectivity dosage and generally the risk of viral infection depends on several factors, such as specifics of the individual (eg. age and health status) and the characteristics of the virus [89]. However, there are no data regarding the detection of enteric viruses in surface waters in the Vhembe District, except in a study done by Obi et al. [9], which detected somatic phages, a likely indicator of viral contamination of water [97,98,99]. Hence, little is known regarding the frequency and pattern of viral contamination of drinking water sources in resource poor settings, such as rural areas in the Vhembe District [100]. Human anthropogenic activities, animals, and agricultural activities may have huge impact on the prevalence of viruses in nonprotected water sources [5]. The river water is potentially hazardous to public health if stored at the household in various types of containers and under questionable hygienic conditions and used untreated [7]. 

## 4. Conclusions

Water samples that were collected from 10 sites in eight rivers in the Limpopo Province were analyzed for faecal contamination as well as human enteric pathogens representing bacterial, protozoan, and viral contamination. The enteric pathogens that were detected from the river water samples analyzed in this study only represented a single time point (either winter or summer season) and, therefore, it is possible that the types of enteric pathogens found could differ if the samples were collected at different periods during the year. Generally, the results indicated the deterioration of water quality in these river catchments, which is used by the rural communities as a source of drinking water during intermittent water supplies to taps or when taps and boreholes run dry or break down. The Madadzhe River was the most contaminated river site (Table 2 and Table 3). Agricultural flow and sewage disposal (Table 1) could be the contributing factors in the contamination of this water site [4]. 

In all cases, the prevalence of enteric bacteria, parasites, and viruses in surface water highlights the importance of assessing the water sources used for domestic purposes for contamination Although this study only reports on a one-year surveillance, there is need for the urgent implementation of improved management strategies of these river catchments by Municipalities in the Limpopo Province for catchment sustainability. 

## Figures and Tables

**Table 1:** Activities seen around the selected river water sites.

River Sampling Site	Description of Observed Activities
Mutale river	Laundry, bathing, car washing, cattle drink from the river
Sambandou river	Laundry, cattle and donkeys drink from the river, animals grazing, people fetch water for construction purposes and other household activities, animal faecal matter around the area
Tshinane river	Laundry, bathing, dumping of chicken feathers and blood
Mutshundudi river	Laundry, bathing, littering
Madadzhe river	Agricultural activities, domestic sewage disposal, car washing
Luvuvhu river (Mhinga site)	Laundry, car washing, swimming, bathing, fishing
Luvuvhu river (Mutoti site)	Laundry, bathing, fishing, car washing, cattle drink from the river, people fetch water for construction purposes
Luvuvhu river (Tshino site)	Cattle grazing, fishing
Nzhelele river	Car washing, people fetch water, swimming, agricultural activities
Dzindi river	Agricultural activities

**Table 2:** Physical parameters and indicator bacteria detected in eight rivers in the Limpopo Province.

River Site	Season	pH	Temp (°C)	TDS (mg/L)	Cond (µS/cm)	DO (mg/L)	Total Coliforms (MPN/100 mL)	*E. coli* (MPN/100 mL)	*C. perfringes* (CFU/100 mL)
Mutale river	Summer	7.842	24.4	35.5	77	9.53	>2420	344.8	16
	Winter	8.008	17.6	29.9	63.3	10.89	1732.9	111.2	5
Sambandou river	Summer	7.703	22.9	30.6	63.1	5.19	>2420	135.4	7
	Winter	7.489	17.8	34.3	71.4	9.62	>2420	12.2	7
Tshinane river	Summer	7.83	23.6	45.1	93.6	9.88	>2420	365.4	9
	Winter	7.776	16.0	44.2	89.3	11.07	1732.9	143.9	0
Mutshundudi river	Summer	7.746	21.1	67.3	134.6	7.09	1986.3	62	12
	Winter	7.735	16.6	46.9	94.35	11.14	>2420	980.4	137
Madadzhe river	Summer	7.218	21.2	34.5	69	2.04	>2420	1299.7	21
	Winter	7.186	20.0	102	205.5	9.16	>2420	2420	>500
Luvuvhu river (Mhinga)	Summer	7.358	26.8	71.3	142.6	7.01	>2420	387.3	21
	Winter	8.101	19.2	80.5	140	12.15	>2420	488.4	7
Luvuvhu river (Mutoti)	Summer	8.192	24.9	64.9	130.3	6.66	1986.3	57.1	0
	Winter	8.375	19.2	77.4	134.4	12.29	980.4	29.2	0
Luvuvhu river (Tshino)	Summer	7.361	28.3	59.2	118.9	5.08	>2420	209.8	9
	Winter	8.308	19.9	69.9	121.6	12.52	>2420	235.9	0
Nzhelele river	Summer	6.975	27.2	142.1	285	4.77	>2420	461.1	21
	Winter	8.381	20.3	348.5	601	11.36	1732.9	435.2	19
Dzindi river	Summer	6.791	28.8	26.8	53.3	5.13	>2420	579.4	35
	Winter	8.421	20.0	70.3	122.2	13.43	1732.9	135.4	9
South African Standards [45]		5.0–9.5	18–24	450–900	400–900	6.0–8.0	0–5	0	0
SANS 241-1 [54]		≥5.0–≤9.5	-	≤1200	≤170 *	-	<10	0	-

* mS/m.

**Table 3:** Pathogenic bacteria, protozoa and virus pathogens isolated in various rivers in the Limpopo province, South Africa.

River	Season	Bacteria										Protozoa		Viruses		
		*Com E. coli*	*aEPEC*	*tEPEC*	*EHEC*	*EAEC*	*EIEC*	*ETEC*	*Vibr*	*Salm*	*Shig*	Crypto Oocysts/10 L	Giardia Cysts/10 L	Cell Culture	PCR (Direct)	PCR (Cell Culture)
Mutale river	Summer	√	√		√	√			√	√						
	Winter	√	√			√			√	√						Entero
Sambandou river	Summer	√	√				√	√		√			1			
	Winter	√	√							√						
Tshinane river	Summer	√	√	√	√				√	√						
	Winter	√	√			√				√		2			Rota	
Mutshundudi river	Summer	√	√	√		√		√	√	√			2			
	Winter	√	√			√		√	√	√					Rota, Entero	
Madadzhe river	Summer	√				√		√	√	√					Adeno	
	Winter	√	√		√	√	√		√	√			62	Reo	Noro GI, Noro GII, Hepatitis A, Entero, Astro, Sapo	Adeno
Luvuvhu river (Mhinga site)	Summer	√	√		√	√	√	√	√	√						
	Winter	√								√					Astro	
Luvuvhu river (Mutoti site)	Summer	√	√		√				√	√						
	Winter	√								√					Rota	
Luvuvhu river (Tshino site)	Summer	√	√	√			√	√	√	√						
	Winter	√	√	√		√			√	√					Entero	
Nzhelele river	Summer	√	√	√	√	√			√	√				Reo		
	Winter	√	√			√			√	√						
Dzindi river	Summer	√	√	√	√	√		√	√	√						
	Winter	√	√					√		√					Rota	

√ = present; Com E. coli = commensal; aEPEC = atypical Enteropathogenic *E. coli*; tEPEC = typical Enteropathogenic *E. coli*; EAEC = Enteroaggregative *E. coli*; EHEC = Enterohaemorrhagic *E. coli*; EIEC = Enteroinvasive *E. coli*; ETEC = Enterotoxigenic *E. coli*; *Vibr* = *Vibrio* spp.; *Salm* = *Salmolella* spp.; *Shig = Shigella* spp.
